# Deubiquitinating enzyme USP30 negatively regulates mitophagy and accelerates myocardial cell senescence through antagonism of Parkin

**DOI:** 10.1038/s41420-021-00546-5

**Published:** 2021-07-21

**Authors:** Wei Pan, Yaowen Wang, Xinyu Bai, Yuehui Yin, Limeng Dai, Hong Zhou, Qin Wu, Yan Wang

**Affiliations:** 1 Key Laboratory of Basic Pharmacology of Ministry of Education and Joint International Research Laboratory of Ethnomedicine of Ministry of Education, Zunyi Medical University, 563000 Zunyi, P. R. China; 2 Department of Cardiology, the Second Affiliated Hospital of Chongqing Medical University, Chongqing Cardiac Arrhythmias Therapeutic Service Center, 400010 Chongqing, P. R. China; 3 Department of Medical Genetics, College of Basic Medical Science, Army Medical University (Third Military Medical University), 400038 Chongqing, P. R. China

**Keywords:** Parkinson's disease, Cellular neuroscience

## Abstract

Cell senescence is associated with age-related pathological changes. Increasing evidence has revealed that mitophagy can selectively remove dysfunctional mitochondria. Overexpression of ubiquitin-specific protease 30 (USP30) has been documented to influence mitophagy and deubiquitination of mitochondrial Parkin substrates. This study was conducted to evaluate the roles of USP30 and Parkin in myocardial cell senescence and mitophagy. Initially, myocardial cells were isolated from neonatal SD rats and subjected to d-gal treatment to induce cell senescence, after which the effects of d-gal on mitochondria damage, ROS production, cell senescence, and mitophagy were assessed. The myocardial cells were infected with lentiviruses bearing overexpression plasmids or shRNA targeting Parkin or USP30 to elucidate the effects of Parkin and USP30 on d-gal-induced mitophagy damage and cell senescence. Finally, aging was induced in rats by subcutaneous injection of d-gal to determine the role of Parkin and USP30 on cell senescence in vivo. d-gal was found to trigger mitochondria damage, ROS production, and cell senescence in myocardial cells. The overexpression of Parkin or silencing of USP30 reduced d-gal-induced mitochondrial damage and relieved d-gal-induced myocardial cell senescence. Moreover, the in vivo experiments validated that either elevation of Parkin or silencing USP30 could alleviate d-gal-induced myocardial cell senescence in rats. Silencing USP30 alleviates d-gal-induced mitochondrial damage and consequently suppresses myocardial cell senescence by activating Parkin. Our study highlights the potential of USP30 as a novel target against myocardial cell senescence.

## Introduction

Cell senescence is a fundamentally irreversible process of growth arrest that occurs when division-competent cells are exposed to various stimuli and stressors [1]. Cells that express markers of senescence have been documented to aggregate at sites of pathology associated with chronic age, highlighting the link between cell senescence and aging and age-related diseases in vivo [2]. Among diseases related to age, cardiovascular diseases account for a large proportion, which are associated with a high risk of mortality and morbidity. The prevalence of heart failure increases with age [3], which may be due to the cumulative loss of myocardial cells due to stress in the heart. Therefore, facilitating myocardial cell regeneration and improving myocardial cell survival have often been the primary targets for heart failure treatment [4].

Mitochondria have been well documented to exert a protective effect on cellular senescence [5]. However, the protective effects of mitochondria decline with age, which is often accompanied by an alteration of mitochondrial morphology [6]. Mitophagy can be understood as a process of the distinct autophagic removal of the mitochondria, aimed at maintaining a steady-state turnover of the mitochondria, which is regulated by different signals, including ubiquitination [7]. Ubiquitin-specific protease 30 (USP30) is a deubiquitinase localized to the mitochondria, which can antagonize mitophagy driven by ubiquitin ligase Parkin and protein kinase PINK1 [8]. More specifically, USP30 has been reported to mediate Parkin and PINK1-dependent mitophagy following the acute depolarization of the mitochondria [9] via the deubiquitylating Parkin substrates in the mitochondria [10]. The overexpression of USP30 has been reported to inhibit the recruitment of Parkin to damaged mitochondria [11]. As an E3-ubiquitin ligase, Parkin is a member of the RING-in-between-RING family, which is fundamentally active due to its autoubiquitination, and has been shown to be involved in a wide array of biological processes, including vesicle trafficking, cell survival pathways, and mitophagy [12]. Parkin is situated in the cytosol but subsequently translocates to the mitochondria for ubiquitination of mitochondrial proteins [13]. The downregulation of Parkin has been implicated with aberrant mitochondrial accumulation in aging myocardial cells [14]. d-galactose (d-gal) administration has often been used to induce a myocardial aging model in previous investigations [15]. Based on the aforementioned exploration of the literature, we designed this study to investigate the underlying regulatory mechanism of USP30 in d-gal-induced mitophagy and myocardial cell senescence in relation to Parkin, with the intention of identifying a conceptual theoretical foundation for an enhanced understanding of cell senescence, specifically in myocardial tissue.

## Results

### d-gal induces mitochondria damage, reactive oxygen species (ROS) production, and cell senescence of myocardial cells in neonatal rats

**Fig. 1:**
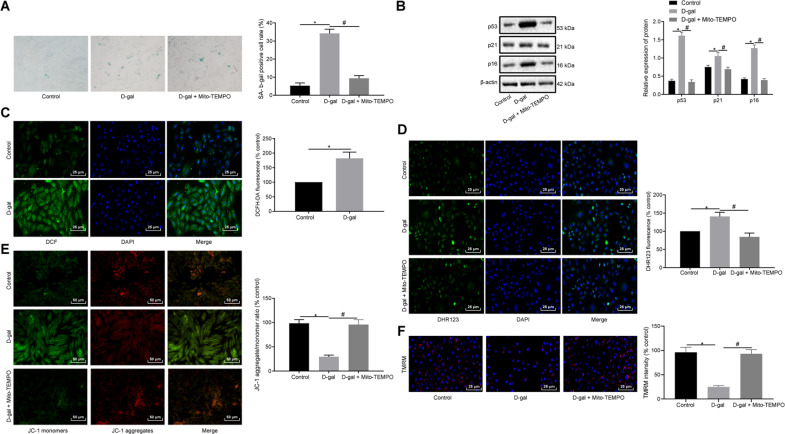
d-gal treatment triggers mitochondria damage, ROS production, and cell senescence in myocardial cells from neonatal rats. Myocardial cells were treated with d-gal and Mito-TEMPO. **A** Representative images and quantitative analysis of cell senescence detected by SA-β-gal staining (×200). **B** Immunoblots of p53, p21, and p16 expression in myocardial cells and densitometric quantification of the relative band intensity. **C** Representative images and quantitative analysis of total ROS in myocardial cells assessed by DCFH-DA immunofluorescence assay (×400). **D** Representative images and quantitative analysis of mitochondrial ROS in myocardial cells examined by DHR123 immunofluorescence assay (×400). **E**, **F** MMP in myocardial cells evaluated by JC-1 immunofluorescence (**E**, ×200) and TMRM (**F**, ×400) assays. *P < 0.05 compared with control myocardial cells. ^#^*P* < 0.01 com*p*ared with the myocardial cells with d-gal treatment. Measurement data are expressed as mean ± standard deviation. Comparisons among multiple groups are analyzed by ANOVA with Tukey’s post hoc test. The experiment is repeated three times.

Accumulating evidence continues to highlight the capacity of d-gal treatment to induce an animal model of aging, and the free radical theory postulates that the oxidative damage caused by ROS plays a significant role in the normal process of aging [16, 17]. To investigate whether d-gal could induce myocardial cell senescence in neonatal rats, myocardial cells were isolated from neonatal SD rats and subjected to d-gal treatment in vitro. Next, Senescence-associated β-galactosidase (SA-β-gal) staining and western blot analysis were conducted to detect cell senescence as well as the expression of senescence-related genes (p53, p21, and p16), the results of which illustrated that d-gal treatment increased SA-β-gal activity as well as the expression of p53, p21, and p16, suggesting that d-gal could induce neonatal myocardial cell senescence (Fig. 1A, B). A DCFH-DA immunofluorescence assay was subsequently conducted to determine the total ROS of the cells, which revealed the increased intensity of DCFH-DA fluorescence in myocardial cells following d-gal treatment (Fig. 1C). Mitochondrial damage has been reported to be a primary source of abnormal ROS levels, with mitochondrial quality control being a key factor in determining various cellular outcomes, including aging [18–20]. DHR123 immunofluorescence assay of mitochondrial ROS indicated that d-gal treatment led to an increase in DHR123 fluorescence intensity (Fig. 1D). Besides, JC-1 staining and TMRM assay revealed that d-gal treatment led to a reduction in mitochondrial membrane potential (MMP) in the myocardial cells. The decrease of MMP induced by the d-gal treatment could be impeded by treatment of mitochondria with Mito-2,2,6,6-tetramethylpiperidinyl-1-oxyl (TEMPO, a specific antioxidant of mitochondrial ROS) (Fig. 1E, F). Moreover, Mito-TEMPO inhibited d-gal-induced mitochondrial ROS production and cell senescence (Fig. 1A, B, D). These results provided evidence verifying that myocardial cells from neonatal rats treated with d-gal exhibited an increase in mitochondrial damage, ROS production, and cell senescence.

### d-gal damages mitophagy to induce cell senescence in myocardial cells

**Fig. 2:**
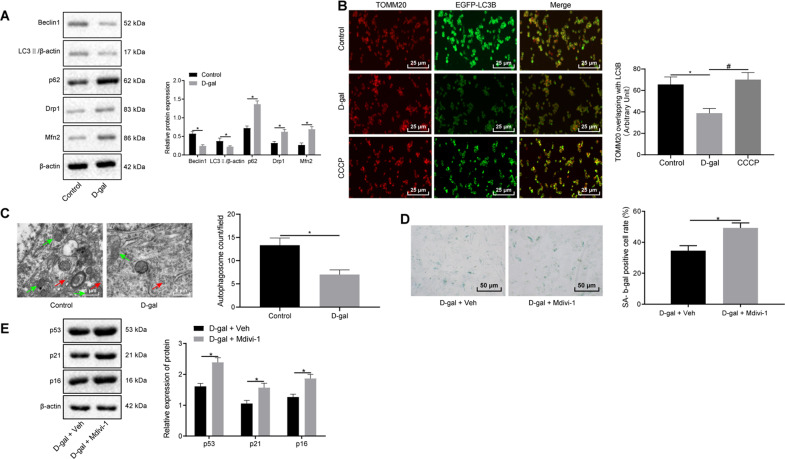
d-gal promotes cell senescence by inducing mitochondrial damage in myocardial cells. Myocardial cells were treated with d-gal, Mdivi-1, and Veh. **A** Immunoblots of mitophagy-related genes (Beclin1, LC3II/β-actin, p62, Drp1, and Mfn2) and densitometric quantification of the relative band intensity. **B** The colocalization of TOMM20-stained mitochondria and EGFP-LC3B detected by immunofluorescence assay (×400). **C** Representative images and quantitative analysis of autophagosomes observed under a TEM (×10,000). Autophagolysosomes are marked with red arrows and mitochondria with black arrows. **D** Representative images and quantitative analysis of cell senescence detected by SA-β-gal staining (×200). **E** Immunoblots of cell senescence-related genes (p53, p21, and p16) and densitometric quantification of the relative band intensity. In panel **A**–**C**, *P < 0.05 compared with the control myocardial cells. In panel **D** and **E**, *P < 0.05 compared with myocardial cells with treatment of d-gal and Veh. Measurement data are expressed as mean ± standard deviation. Comparisons between two groups are analyzed by non-paired *t* test. The experiment is repeated three times.

Mitochondrial autophagy represents specific autophagy, which has been described as a dynamic process [7]. We next set out to detect the expression of the autophagy proteins LC3 and p62 in myocardial cells. Besides, dynamin-related protein (Drp1) and mitofusin 2 gene (Mfn2) are known to be proteins involved in mitochondrial dynamics that are associated with mitochondrial autophagy [21, 22], so we examined Drp1 and Mfn2 expression. Western blot analysis revealed that d-gal treatment decreased the expression of Beclin1 and the ratio of LC3-II/β-actin, while increasing expression of p62, Drp1, and Mfn2 (Fig. 2A). The myocardial cells were then transfected with EGFP-LC3B to label autophagy. The colocalization of mitochondrial membrane marker TOMM20-stained mitochondria and EGFP-LC3B were indicative of the formation of an autophagosome. We found that treatment with d-gal led to a decrease in the number of cells with the colocalization of TOMM20-stained mitochondria and EGFP-LC3B, while the number of cells with this colocalization in the CCCP group failed to exhibit a significant difference from that in the control group (Fig. 2B). The ultrastructural analysis of mitochondrial autophagy using transmission electron microscopy (TEM) has been reported to be a direct and effective method to confirm mitochondrial autophagy or clearance [23]. Observation under TEM revealed that the mitochondria were damaged, with the number of autophagosomes reduced following d-gal treatment (Fig. 2C). In addition, we treated the cells with the mitochondria autophagy inhibitor Mdivi-1. The results suggested that treatment with both d-gal and Mdivi-1 led to increased SA-β-gal activity and expression of p53, p21, and p16 relative to the treatment of d-gal alone (*P* < 0.05; Fig. 2D, E). Taken together, the aforementioned findings demonstrated that the d-gal treatment could inhibit mitophagy and promote cell senescence in myocardial cells.

### Parkin overexpression reduces d-gal-damaged mitophagy and alleviates cell senescence

**Fig. 3:**
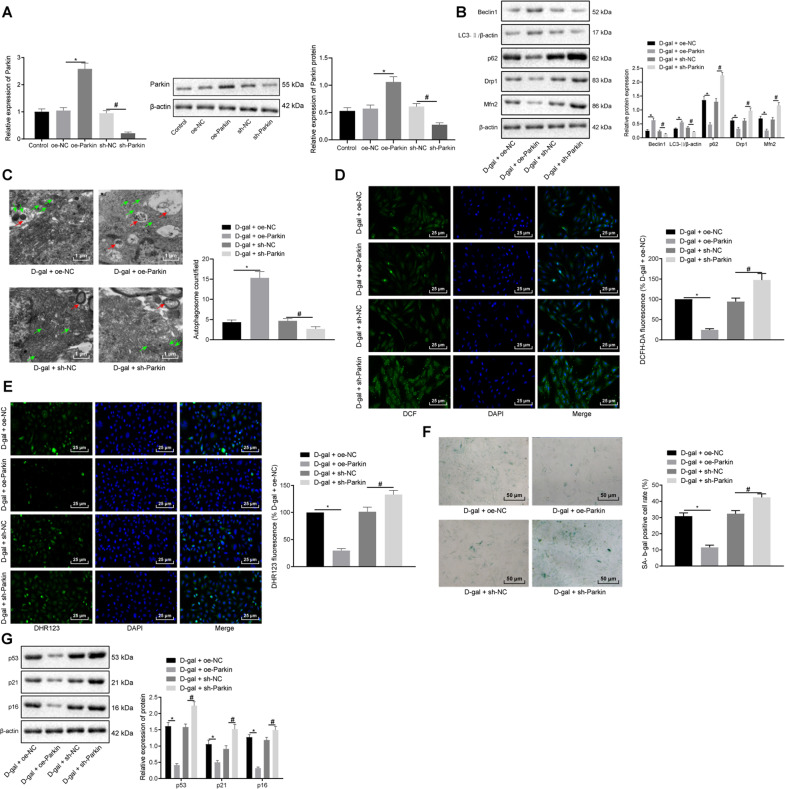
Upregulated Parkin contributes to the attenuation of d-gal-induced mitochondrial damage and cell senescence. Myocardial cells were treated with d-gal and infected with lentivirus of oe-Parkin or sh-Parkin. **A** RT-qPCR and Western blot analysis of Parkin expression. *P < 0.05 compared with the transduction of oe-NC. ^#^*P* < 0.05 com*p*ared with the transduction of sh-NC. **B** Immunoblots of mitophagy-related genes (Beclin1, LC3II/β-actin, p62, Drp1, and Mfn2) and densitometric quantification of the relative band intensity. **C** Representative images and quantitative analysis of autophagosomes observed under a TEM (×10,000). Autophagolysosomes are marked with red arrows and mitochondria with black arrows. **D** Total ROS in myocardial cells assessed by DCFH-DA immunofluorescence assay (×400). **E** Representative images and quantitative analysis of mitochondrial ROS in myocardial cells examined by DHR123 immunofluorescence assay (×400). **F** Representative images and quantitative analysis of cell senescence detected by SA-β-gal staining (×200). **G** Immunoblots of p53, p21, and p16 in myocardial cells and densitometric quantification of the relative band intensity. In panels **B**–**G**, *P < 0.05 compared with the treatment of d-gal + oe-NC. ^#^*P* < 0.05 compared with the treatment of d-gal and sh-NC. Measurement data are expressed as mean ± standard deviation. Comparisons among multiple groups are analyzed by ANOVA with Tukey’s post hoc test. The experiment is repeated three times.

Stress-induced mitochondrial membrane depolarization has been reported to stabilize Pink1, triggering the recruitment of Parkin (a kind of E3-ubiquitin ligase) to the mitochondria [9]. Parkin-mediated ubiquitination of mitochondrial substrate is involved in mitophagy and cell senescence [10]. Therefore, we set out to ascertain the role of Parkin in d-gal-regulated mitophagy and cell senescence, and myocardial cells were infected with lentiviruses containing oe-Parkin or sh-Parkin. The results indicated that oe-Parkin triggered heightened levels of Parkin expression, while suggesting that sh-Parkin led to its poor expression, which validated its infection efficiency (Fig. 3A). Furthermore, sh-Parkin showed the best interfering efficiency compared with sh-Parkin-1 and sh-Parkin-2, leading us to choose sh-Parkin for further silencing of Parkin (Supplementary Fig. 1). Western blot analysis revealed that in the presence of d-gal, overexpressing Parkin elevated the expression of Beclin1 and the ratio of LC3-II/β-actin, while decreasing the expression of p62, Drp1, and Mfn2. On the other hand, the silencing of Parkin led to opposite results in the myocardial cells (Fig. 3B). Observation under TEM revealed that the number of mitochondrial autophagosome was increased by the transduction of oe-Parkin together in the presence of D-gal, and decreased by Parkin gene silencing in the presence of d-gal (Fig. 3C). Moreover, DCFH-DA and DHR123 immunofluorescence assays were employed to evaluate ROS, which revealed that in the presence of d-gal, overexpressed Parkin resulted in a reduction in DCFH-DA and DHR123 fluorescence intensity while silencing of Parkin led to opposite effects (Fig. 3D, E). Furthermore, SA-β-gal staining and western blot analysis suggested that SA-β-gal activity and expression of p53, p21, and p16 were decreased following the overexpression of Parkin and were increased following the silencing of Parkin after treatment with d-gal (Fig. 3F, G). Taken together, the overexpression of Parkin could alleviate damaged mitophagy and cell senescence induced by d-gal.

### USP30 negatively regulates mitophagy and promotes myocardial cell senescence through antagonism on Parkin

**Fig. 4:**
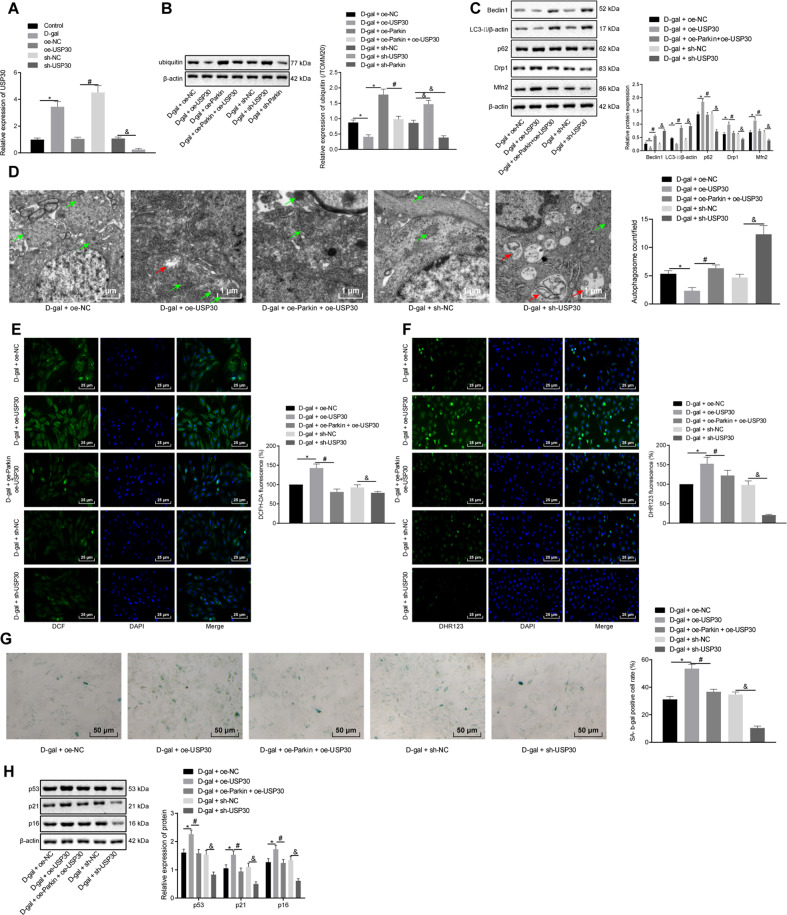
USP30 promotes d-gal-induced mitochondrial damage and accelerates cell senescence. Myocardial cells were introduced with both d-gal and oe-Parkin, oe-USP30, sh-USP30, or sh-Parkin. **A** RT-qPCR analysis of USP30 expression. *P < 0.05 compared with control. ^#^*P* < 0.05 com*p*ared with the introduction of oe-NC. ^&^*P* < 0.05 compared with the introduction of sh-NC. **B** Immunoblots of ubiquitin in mitochondria of myocardial cells and densitometric quantification of the relative band intensity. **C** Immunoblots of mitophagy-related genes (Beclin1, LC3II/β-actin, p62, Drp1, and Mfn2) and densitometric quantification of the relative band intensity. **D** Representative images and quantitative analysis of autophagosomes observed under a TEM (×100,00). Autophagolysosomes are marked with red arrows and mitochondria with black arrows. **E** Representative images and quantitative analysis of total ROS in myocardial cells detected by DCFH-DA immunofluorescence assay (×400). **F** mitochondrial ROS in myocardial cells assessed by DHR123 immunofluorescence assay (×400). **G** Representative images and quantitative analysis of cell senescence detected by SA-β-gal staining (×200). **H** Immunoblots of p53, p21, and p16 expression in myocardial cells and densitometric quantification of the relative band intensity. In panels **B**–**H**, *P < 0.05 compared with the treatment of D-gal + oe-NC. ^#^*P* < 0.05 compared with the treatment of d-gal + oe-Parkin. ^&^*P* < 0.05, compared with the treatment of d-gal + sh-NC. Measurement data are expressed as mean ± standard deviation. Comparisons among multiple groups are analyzed by ANOVA with Tukey’s post hoc test. The experiment is repeated three times.

Next, to further elucidate whether the function of Parkin in d-gal-induced mitochondrial damage and d-gal-promoted cell senescence was associated with USP30, we first determined the expression of USP30, finding that d-gal treatment increased USP30 expression (Fig. 4A). Next, the expression of USP30 was elevated or silenced in the myocardial cells via the introduction of a lentivirus. Reverse transcription-quantitative polymerase chain reaction (RT-qPCR) results revealed that the introduction of oe-USP30 increased USP30 expression, while the introduction of sh-USP30 decreased its expression (Fig. 4A), among which sh-USP30 revealed superior interfering effects compared to sh-USP30-1 and sh-USP30-2 (Supplementary Fig. 2). Therefore, lentivirus-based sh-USP30 was selected for subsequent experiments. Western blot analysis showed the mitochondrial expression of ubiquitin was reduced after the introduction of d-gal + oe-USP30 or d-gal + sh-Parkin. Meanwhile, ubiquitin was increased after the introduction of d-gal + oe-Parkin as well as d-gal + sh-USP30 (Fig. 4B). Western blot analysis displayed that the introduction of oe-USP30 led to a reduction in the expression of Beclin1, reduced the ratio of LC3-II/β-actin, but increased the expression of p62, Drp1, and Mfn2. Conversely, the introduction of sh-USP30 or oe-Parkin and oe-USP30 caused opposite effects in myocardial cells after treatment of d-gal (Fig. 4C). Moreover, observation under TEM revealed that the introduction of oe-USP30 decreased the number of autophagosomes, while USP30 downregulation or Parkin elevation increased the number of autophagosome in the presence of d-gal (Fig. 4D). Furthermore, immunofluorescence assay of ROS demonstrated that DCFH-DA and DHR123 fluorescence intensity were strengthened following the introduction of oe-USP30 and weakened following the introduction of sh-USP30 or oe-Parkin and oe-USP30 in the presence of d-gal (Fig. 4E, F). In addition, SA-β-gal staining and western blot analysis indicated that the introduction of oe-USP30 elevated SA-β-gal activity and expression of p53, p21, and p16, while the introduction of sh-USP30 or oe-Parkin and oe-USP30 caused opposite results after d-gal treatment (Fig. 4G, H). These results demonstrated that USP30 could promote d-gal-induced mitochondrial damage and cell senescence through antagonizing Parkin.

### Parkin overexpression or USP30 silencing alleviates d-gal-induced myocardial cell senescence in rats

**Fig. 5:**
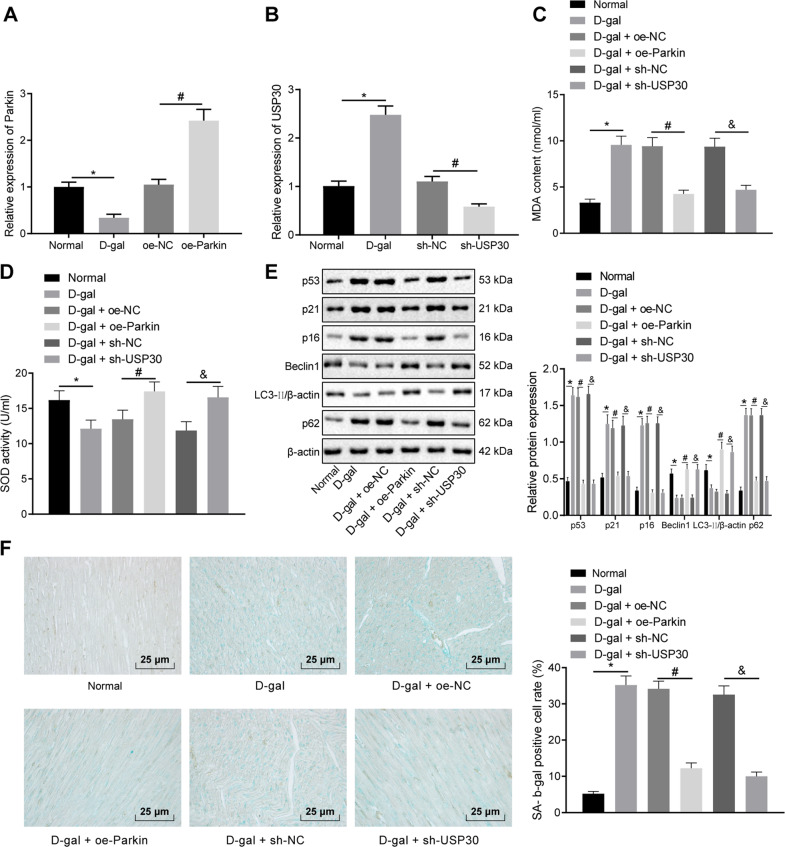
Parkin elevation or USP30 downregulation attenuates d-gal-induced myocardial cell senescence in a rat model of aging. Rats are injected with d-gal, oe-Parkin and sh-USP30. **A** RT-qPCR analysis of Parkin expression in rat myocardial tissues. *P < 0.05 compared with the normal rats. ^#^*P* < 0.05 com*p*ared with the injection of oe-NC. **B** RT-qPCR analysis of USP30 expression in rat myocardial tissues. *P < 0.05 compared with the normal rats. ^#^*P* < 0.05 compared with the injection of sh-NC. **C** Quantitative analysis for MDA content in rat serum. **D** Quantitative analysis for SOD activity in rat serum. **E** Immunoblots of p53, p21, p16, p62, Beclin1, and LC3II/β-actin in rat myocardial tissues and densitometric quantification of the relative band intensity. **F** Representative images and quantitative analysis of cell senescence detected by SA-β-gal staining (×400). In panels **C**–**F**, *P < 0.05 compared with the normal rats. ^#^*P* < 0.05, compared with the injection of d-gal and oe-NC. ^&^*P* < 0.05 compared with the injection of d-gal and sh-NC. Measurement data are expressed as mean ± standard deviation. Comparisons among multiple groups are analyzed by ANOVA with Tukey’s post hoc test. n = 8.

Finally, to determine whether USP30 and Parkin could influence myocardial cell senescence in vivo, rats were treated with d-gal to induce cellular aging. RT-qPCR results indicated that the treatment of oe-Parkin increased Parkin expression and the treatment of sh-USP30 decreased the USP30 expression in the d-gal-induced rat aging model (Fig. 5A, B). Assays of the malondialdehyde (MDA) content and superoxide dismutase (SOD) activity revealed that treatment with d-gal elevated MDA content and weakened SOD activity, while the treatment of oe-Parkin as well as sh-USP30 caused opposite effects in the presence of d-gal (Fig. 5C, D). Moreover, western blot analysis demonstrated that treatment with d-gal triggered an elevation in the expressions of p53, p21, p16, and p62 and a reduction in expression of Beclin1 and ratio of LC3-II/β-actin, while treatment with oe-Parkin as well as d-gal and sh-USP30 resulted in opposite results in the presence of d-gal (Fig. 5E). Meanwhile, SA-β-gal activity was increased after treatment with d-gal, and decreased after the treatment with d-gal and oe-Parkin as well as d-gal and sh-USP30 (Fig. 5F). Taken together, the overexpression of Parkin or silencing of USP30 could relieve d-gal-induced myocardial cell senescence in vivo.

## Discussion

Cell senescence is a biological event implicated in various processes, including aging and tissue repair [1]. Over the past few years, mitochondrial dysfunction has been linked with major aging-related phenotypes [6]. Accumulating evidence has been presented suggesting that deubiquitinase USP30 plays an inhibitory role in mitophagy through functioning against ubiquitination mediated by Parkin [11]. This study clarified the functions of USP30 on d-gal-regulated mitophagy and myocardial cell senescence. Conjointly, our findings demonstrated that USP30 silencing could alleviate d-gal-induced mitophagy and myocardial cell senescence by releasing the inhibition of Parkin.

Initially, d-gal was observed to induce mitochondrial damage, ROS generation, cell senescence, and mitophagy in myocardial cells, as reflected by increased SA-β-gal activity and expression of p53, p21, p16, p62, Drp1, and Mfn2 as well as reduced MMP and expression of Beclin1 and ratio of LC3-II/β-actin. d-gal has been reported to act via induction of mitochondrial enzymes and DNA damage in rats [24]. Furthermore, d-gal-induced HepG2 cell apoptosis is correlated with mitochondria-related pathways [25]. The regulation of mitochondrial quality control and dynamics has been reported to alleviate d-gal-induced fulminant hepatic failure [26]. Meanwhile, d-gal contributes to the generation of ROS and cell death of cultured hepatocytes [27]. Broadly speaking, the generation of ROS has been implicated in the process of senescence [16]. In addition, cell senescence has been revealed to be regulated through the p53/p21/p16 signaling axis [28]. Another study suggested that the p53/p21 and p16/pRB pathways were related to cell senescence under the regulation of SWI/SNF complex subunits [29]. In addition, p62 has been highlighted as a multidomain protein linked to autophagy, apoptosis, and cell death [30]. Drp1 and Mfn2, with their distinct interacting protein network, can contribute to the maintenance of the mitochondrial fission–fusion balance [31], dysregulation of which can lead to the perturbation of mitochondrial dynamics. Thus, our findings unveiled that d-gal facilitated mitochondria damage, ROS production, and cell senescence in myocardial cells.

In addition, an accompanying notable finding of our study is that silencing USP30 could relieve d-gal-induced mitochondrial damage and alleviate d-gal-induced cell senescence by enhancing ubiquitination regulated by Parkin in myocardial cells. Mitophagy plays a vital role in controlling the quality of the mitochondria through the elimination of impaired mitochondria [32], which exerts a functional influence on the development of cardiovascular diseases such as heart failure and myocardial ischemic injury [33]. Both USP30 and Parkin have been implicated in the homeostatic mediation of untypical ubiquitin chains on mitochondria [34]. In most cells, overexpression of heterologous Parkin has been shown to result in a distinct elevation of mitochondrial clearance [10]. Existing literature has suggested that Parkin plays a crucial role in the elimination of impaired mitochondria in myocardial cells under stress, the absence of which would lead to an aggregation of abnormal mitochondria in aging myocardial cells [14]. Parkin has also been identified to contribute to the adaptation to stress in the myocardium by clearing damaged mitochondria [35]. Furthermore, the presence of Parkin has been reported to aid in the attenuation of microtubule senescence in dopaminergic neurons [36]. Parkin E3-ubiquitin ligase activity is known to be essential for mitochondria since it can effectively eliminate dysfunctional mitochondria [37, 38]. The extensive activation of the ubiquitin–proteasome system by Parkin is essential for mitochondrial phagocytosis, whereby Parkin activates the ubiquitin–proteasome system to extensively degrade outer mitochondrial membrane proteins [39]. In mammals, Mfn1 and Mfn2 are poly-ubiquitinated and degraded in response to the activation of Parkin [40, 41]. However, degradation of these mitochondrial proteins may be involved in the isolation of dysfunctional mitochondria, thereby enhancing the functional benefits of mitochondria [42, 43]. Thus, the results of the current study highlighted a potential relationship between a decrease in Mfn2 and Drp1 with the proteasome pathway. Meanwhile, deficiency of USP30 in cells overexpressing Parkin facilitates the elimination of mitochondria under the intervention of mitochondrial depolarizing agents [9]. Our findings provided further verification explaining the functions of Parkin and USP30 in mitophagy and cell senescence, demonstrating that USP30 knockdown could alleviate d-gal-induced mitochondrial damage, and d-gal-promoted myocardial cell senescence by increasing the activity of Parkin.

**Fig. 6:**
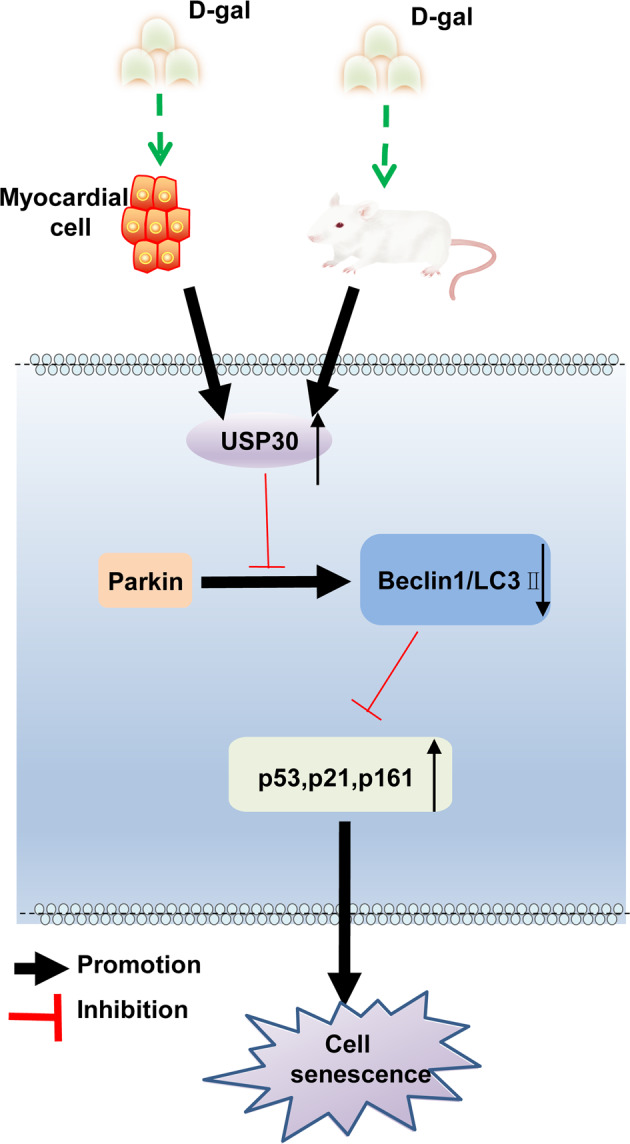
The mechanism graph of the regulatory network and function of USP30. In a d-gal-induced myocardial cell senescence model, overexpression of USP30 inhibits Parkin to decrease expression of Beclin1 and LC3II and to increase expression of p53, p21, and p16, thus suppressing mitophagy, promoting ROS production and myocardial cell senescence.

In conclusion, the findings of the present study demonstrated that silencing USP30 upregulated Parkin, resulting in the stimulation of mitophagy and deceleration of myocardial cell senescence (Fig. 6). These findings provide fresh insights into a promising novel approach for future treatments of cardiovascular diseases and heart failure. However, this research is still at a preclinical stage, and the role and mechanism of USP30 in myocardial cell senescence have yet to be investigated using clinical samples. Therefore, future investigations with a large cohort of clinical samples should be included in future research to determine the underlying mechanism of USP30 and Parkin in myocardial cell senescence.

## Materials and methods

### Primary culture of myocardial cells

New-born myocardial cells were obtained from Sprague-Dawley (SD) rats (aged 1–2 days) that were acquired from the animal research center of Zunyi Medical University. The heart tissues were collected in accordance with previously reported methods [44]. The rat left ventricle was cut into pieces, trypsinized, and incubated in Dulbecco’s modified Eagle’s medium (DMEM) (Sigma-Aldrich, St. Louis, MO, USA) supplemented with 20% fetal bovine serum (FBS) and 100 U/mL penicillin and streptomycin (Sigma-Aldrich, St. Louis, MO, USA) in a 50-mL Corning cell culture flask for 1.5 h at 37 °C with 5% CO_2_. The un-attached cells were subsequently inoculated into a six-well plate (5–6 × 10^5^ cells/ mL) at 37 °C with 5% CO_2_. After 48 h of incubation, 80% of the cells adhered to the plate and were collected for subsequent experiments.

### Myocardial cell treatment

The myocardial cells in the logarithmic growth phase were inoculated into a six-well plate at the density of 2 × 10^5^ cells/well. When cell confluence reached 30%, cell infection was performed. Specifically, a total of 2 × 10^6^ TU corresponding lentivirus and 5 μg of Poly-brene were added to 1 mL of serum-free and antimicrobial medium. After mixing, the cells were infected and observed under an inverted fluorescence microscope for a period of 2–3 days. Next, 1 μg/mL of puromycin was added to each well in order to screen the stably transfected cells, after which the cells were collected for subsequent experimentation. Overexpression of the vectors of Parkin or USP30 gene were pEGFP-N1 vectors (Clontech, Palo Alto, CA, USA), while pSIH1-H1-copGFP vectors (Cat. # SI501A-1, SBI, USA) were applied as silencing vectors of Parkin or USP30 gene. Meanwhile, the lentivirus-mediated vectors were established by GenePharma (Shanghai, China). A cell senescence model was induced via d-gal treatment, whereby myocardial cells were incubated in DMEM containing d-galactose (5 g/L), mitochondrial division inhibitor (Mdivi-1) (M0199, Sigma-Aldrich, St. Louis, MO, USA), and 100 mM mitochondria Mito-TEMPO (ALX-430-150, Enzo Life Sciences, Raamsdonksveer, The Netherlands) or 10 μM CCCP (catalog number C2759; Sigma-Aldrich) for 48 h. Cells were treated with d-gal, Mito-TEMPO, vehicle (Veh), Mdivi-1, lentivirus-mediated overexpression vector of Parkin (oe-Parkin), overexpression vector of USP30 (oe-USP30), short hairpin RNA against Parkin (sh-Parkin, sh-Parkin-1, sh-Parkin-2) or short hairpin RNA against USP30 (sh-USP30, sh-USP30-1. sh-USP30-2), or their matched negative control (NC), alone or in combination.

### Immunofluorescence assay

Immunofluorescence assay was performed based on a previously reported method [45]. Myocardial cells overexpressing EGFP-LC3B were fixed with 4% paraformaldehyde for 15 min and permeabilized by 0.03% Triton X-100 (160-24751, Wako Pure Chemical Industries, Ltd., Tokyo, Japan) for 60 min. Next, the cells were sealed with 0.1% bovine serum albumin (BSA) (A2153, Sigma-Aldrich, St. Louis, MO, USA) for 60 min and incubated with the primary antibody TOMM20 (ab220822, Abcam Inc., Cambridge, MA, USA) as well as a secondary antibody against immunoglobulin G (IgG) (ab150083, Abcam Inc., Cambridge, MA, USA) in accordance with the instructions listed in their respective manuals. The cells were subsequently observed and photographed under a confocal laser scanning microscope (LSM510, Carl Zeiss, Jena, Germany). Subsequently, dichloro-dihydro-fluorescein diacetate (DCFH-DA) and dihydrorhodamine 123 (DHR123) immunofluorescence assays were conducted as per the provided instructions. Myocardial cells were seeded into a 96-well microtest plate at the density of 3 × 10^4^ cells/well (237105, Thermo Fisher Scientific, Waltham, MA, USA). DCFH-DA (STA-342, Cell Biolabs, Inc., San Diego, CA, USA) was utilized to measure the reactive oxygen species (ROS) of total cells. After the cells had been incubated with 10 mM DCFH-DA at 37 °C for 10 min, nuclear staining was performed with the addition of 4,6-diamino-2-phenyl indole (DAPI), which was then removed by PBS washing (three washes in total). A fluorescence microplate (Infinite F200, Tecan Japan, Kanagawa, Japan) was employed to detect DCF fluorescence at an excitation wavelength of 485 nm and an emission wavelength of 535 nm. DHR 123 staining was conducted to analyze ROS production. The fluorescence microscope (Olympus BX60, Tokyo, Japan) was applied for photographing and observation prior to nuclear staining with DAPI.

### JC-1 staining for MMP detection

MMP was detected using the JC-1 method. A lipotropism positive ion probe JC-1 with formed J-aggregation was employed to detect MMP in accordance with the instructions provided in the manual (Molecular Probes, Eugene, OR, USA). The cells were then cultured in a glass dish (SPL Life Sciences Co., Ltd., Pochoen, Korea). JC-1 was subsequently dissolved in 1 mg/mL dimethyl sulfoxide (DMSO) and diluted in serum-free medium (final concentration, 1 μg/mL), which was then added into the cells for a 10-min period incubation at 37 °C. The cells were then incubated in 1 mL of culture medium and analyzed under a fluorescence microscope (Olympus, Tokyo, Japan).

In addition, MMP was measured using the fluorescent tetramethylrhodamine methyl ester (TMRM, 250 nM, Invitrogen, USA). TMRM was then added to the cells and incubated at 37 °C for 20 min. After incubation, the cells were washed several times and observed under an inverted confocal microscope (IX81, Olympus) with analysis on images by ImageJ (https://imagej.nih.gov/ij/).

### TEM

The cells were fixed in 2.5% glutaraldehyde solution at 4 °C for 4 h, rinsed four times using 0.1 M phosphate buffer saline (PBS) (15 min each), and fixed with 1% osmic acid solution at 4 °C for 2 h. The cells were subsequently dehydrated using 50, 70, 90, and 100% gradient ethanol (15 min each) and permeabilized (100% acetone: resin = 1:1 for 2 h, 100% acetone: resin = 1:2 for 2 h, pure resin overnight). The cells were then embedded in Epon812 resin, aggregated in an oven (37°C for 12 h, 45°C for 12 h, and 60°C for 48 h), sectioned into ultrathin slices (thickness, 60–70 nm), stained with uranyl acetate and lead nitrate and observed under a TEM (FEI Tecnai G2 Spirit Bio TWIN, Thermo Fisher Scientific, Waltham, MA, USA). Five visual fields were randomly selected to observe and quantify the number of autophagic lysosomes. The experiment was repeated three times.

### d-gal-induced rat model with aging

A total of 80 SD rats (aged 3–4 weeks, weighing 200–230 g, purchased from the animal research center of Zunyi Medical University) were acclimated for 1 week with free access to food and water and a 12-h light/darkness cycle. The rats were then randomly placed into ten groups (*n* = 8 each): normal, oe-NC, oe-Parkin, sh-NC, sh-USP30, d-gal, d-gal + oe-NC, D-gal + oe-Parkin, d-gal + sh-NC, and d-gal + sh-USP30 groups. d-gal was used to induce aging in the rats. From the first week, the rats were subcutaneously injected with 150 mg/kg d-gal every day for 10 consecutive weeks. On the fourth week of d-gal treatment, the rats were infected with lentiviruses containing oe-Parkin or sh-USP30 (1 × 10^7^ TU/30 µL/rat) by intracardiac injection, followed by feeding for consecutive 6 weeks. The rats were subsequently euthanized using 1% pentobarbital sodium after d-gal treatment, with samples of myocardial tissue and blood collected for evaluation. The SOD activity in the rat serum was identified in accordance with the instructions of the kit (Beyotime Biotechnology Co., Ltd., Shanghai, China). MDA content was determined using an MDA reagent kit (MAK085, Sigma-Aldrich, St. Louis, MO, USA).

### RT-qPCR

Trizol RNA extracting solution (Invitrogen, Carlsbad, CA, USA) was used to extract the total RNA from the myocardial cells or tissues. Reverse transcription was performed in accordance with the specifications of Primescript^TM^ RT reagent Kit (RRO37A, TaKaRa, Tokyo, Japan). A fluorescence quantitative PCR instrument was utilized to amplify the target gene as well as the internal reference. β-actin was regarded as the internal reference. The 2^-ΔΔCt^ method was applied to calculate the relative expression of the target genes [46]. The experiment was repeated three times. Primer sequences are depicted in Table 1.

**Table 1:** Primer sequences for RT-qPCR.

Gene	Primer sequence
Parkin	F: 5’-CTTGGCTACTCGCTGCCGTGTGT-3’
	R: 5’-TACCTGTTGTACTGCTCTTCTCC-3’
USP30	F: 5’-AGTCACTTGCCACACGAGAG-3’
	R: 5’-CCCAAGTGGCAGCTGGAATA-3’
β-actin	F: 5’-TGGAATCCTGTGGCATCC-3’
	R: 5’-TCGTACTCCTGCTTGCTG-3’

*RT-qPCR* reverse transcription-quantitative polymerase chain reaction, *F* forward, *R* reverse.

### Western blot analysis

The mitochondria and cytoplasm were obtained using a specific kit (89874, Thermo Fisher Scientific, Waltham, MA, USA). Next, the cells were rinsed with precooled PBS and lysed using Radio-Immunoprecipitation assay (RIPA) cell lysis buffer containing protease inhibitor on ice. After centrifugation at 4 °C and 12,000 r/min for 5 min, the supernatant was collected. The bicinchoninic acid (BCA) method was applied to determine the protein concentration. Next, 50 μg of protein was separated by 10% sodium dodecyl sulfate-polyacrylamide gel electrophoresis (SDS-PAGE), transferred onto a nitrocellulose membrane, and blocked with 5% skim milk at room temperature for 2 h. The membrane was then incubated with primary antibodies against Parkin (#4211, 1:1000), p53 (#2524, 1:1000), p21 (ab188224, 1:1000), p16 (ab51243, 1:1000), Beclin1 (ab207612, 1:2000), LC3B (ab48394, 1:1000), p62 (ab56416, 1:1000), Ubiquitin (ab7780, 1:1000), TOMM20 (ab186734, 1:1000), Drp1 (ab184247, 1:1000), Mfn2 (ab101055, 1:1000), and β-actin (ab8226, 1:1000) at 4 °C overnight. The membrane was then incubated with horseradish peroxidase (HRP)-labeled goat-anti mouse or goat-anti-rabbit IgG antibody (ab205719 or ab6721, 1:5000) at 37 °C for 1 h. Parkin and p53 antibodies were purchased from Cell Signaling Technology (Danvers, MA, USA), while the other antibodies were purchased from Abcam Inc. (Cambridge, MA, USA). The membrane then underwent a reaction with enhanced chemiluminescence (ECL) solution (ECL808-25, Biomiga Inc, San Diego, USA) at room temperature for 1 min. An X-ray plate developer (36209ES01, Shanghai Qian Chen BioScience & Technologies Co., Ltd., Shanghai, China) was employed to develop the membrane. With β-actin considered as the internal reference, the gray value ratio between the target band and internal band was regarded as relative expression level. The experiment was repeated three times.

### SA-β-gal staining

The myocardial cells cultured in a 24-well plate were collected for SA-β-gal staining in accordance with the instructions of the kit (K802-250, BioVision, Milpitas, CA, USA). Fresh myocardial tissues were subsequently extracted from the rats and swiftly frozen in liquid nitrogen. The tissues were then sectioned by Leica CM1900 freezing microtome (Leica, Wetzlar, Germany) into slices with at a thickness of 10 μm. The slices were then washed with PBS and incubated with SA-β-gal staining solution (Beyotime Biotechnology Co., Ltd., Shanghai, China) at 37 °C overnight. On the second day, the slices were washed again with PBS and photographed under a BX51 Olympus microscope (Olympus, Tokyo, Japan).

### Statistical analysis

All data were analyzed using a Statistic Package for the Social Science (SPSS) 21.0 statistical software (IBM Corp. Armonk, NY, USA). Measurement data were summarized by the mean ± standard deviation. Comparisons between two groups were analyzed by non-paired *t* test, while comparisons among multiple groups were analyzed by one-way analysis of variance (ANOVA) with a Tukey’s post hoc test. A value of *P* < 0.05 was considered to be indicative of statistical significance.

## Supplementary information

Figure S1

Figure S2
