# Supplementary figures and images for: Deubiquitinating enzyme USP30 negatively regulates mitophagy and accelerates myocardial cell senescence through antagonism of Parkin
Source: Cell Death Discov. 2021 Jul 21;7:187. doi: 10.1038/s41420-021-00546-5 (PMC8295395; doi:10.1038/s41420-021-00546-5)

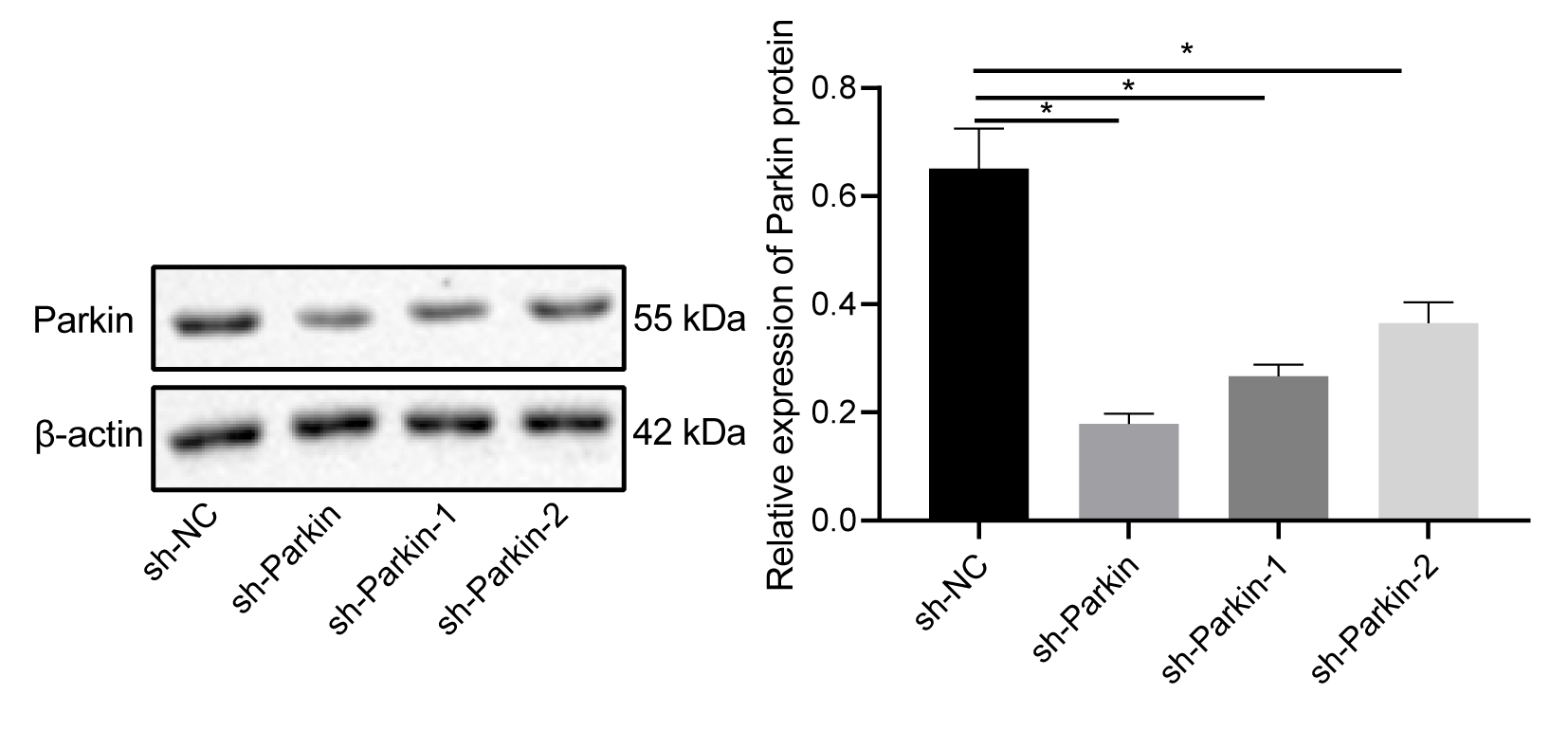

Supplement: Supplementary file 1 — Figure S1 [file 41420_2021_546_MOESM1_ESM.tif]

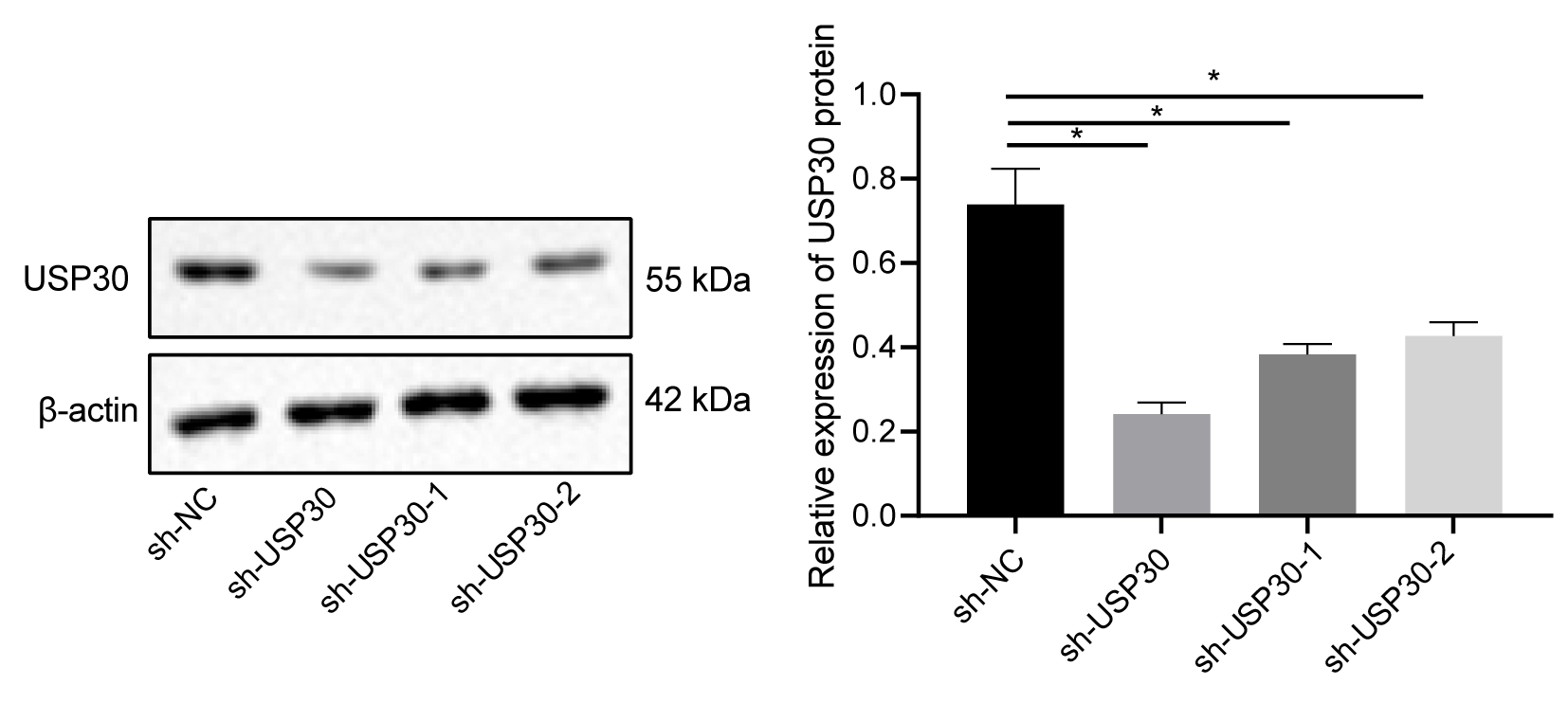

Supplement: Supplementary file 2 — Figure S2 [file 41420_2021_546_MOESM2_ESM.tif]
